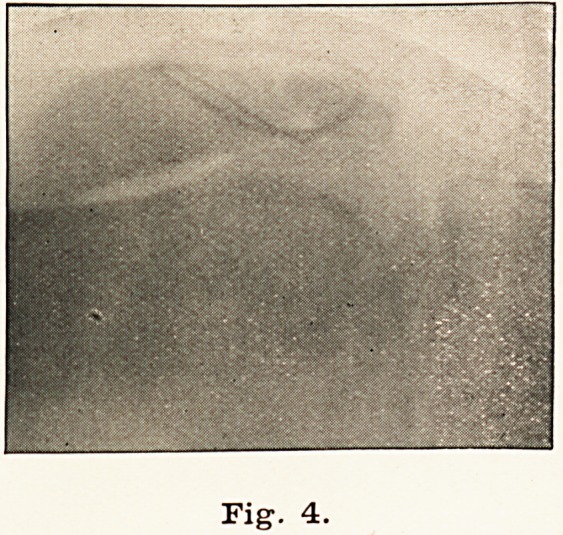# Fracture of the Patella

**Published:** 1899-06

**Authors:** A. W. Prichard

**Affiliations:** Surgeon to the Bristol Royal Infirmary.


### FRACTURE OF THE PATELLA.

BY

A. W. PRICHARD, M.R.C.S.,
*Surgeon to the Bristol Royal Infirmary.*

In a case of fractured patella there is often some difficulty in knowing what treatment it is best to recommend to the patient and his friends, and there are many points to be considered. I do not mean to go into all details; these are best left to the judgment of the surgeon in charge of the case. I will briefly mention the five methods of treatment that are now in fashion, and offer some remarks on each.

Firstly, the plan without operation. This, the old one, consists of rest with the application of splint and bandages. There are various excellent ways of drawing the fragments together, and perhaps in almost the majority of cases this method is the one to be recommended, and it often gives fair results; but the best result that can be hoped for is a ligamentous union with more or less impairment of extension, and the probability is that the individual will never be able to run or climb a ladder.

Secondly, subcutaneous silk ligature, by surrounding the knee-cap either laterally or vertically by a stout silk cord. In the latter case the ligature is passed by means of a strong needle right into the joint, and is left in, resting on the condyles. I have had no personal experience of this plan, but I cannot imagine that it would be successful in many cases when the tilting of the fragments and the indipping of the fibrous covering are considered. The tilting is present in most cases, as seen in Fig. 1, and is sometimes remarkable, as in one case which I wired, where the fractured surface of the upper fragment was directly forwards. The dipping in of the fibrous covering has been present and has given some trouble in all cases that I have seen operated on.

Thirdly, the recently recommended method of massage of the thigh muscles, and passive movement of the knee from the commencement of the case. This is advocated by those who think that the cause of bad results in cases that have not been operated on is to some extent atrophy of the quadriceps, and contraction of the fasciæ from want of use. I cannot say anything about this treatment from my own experience—I want to see it tried—but it can never result in bony union if the fragments have been separated, and a ligamentous union can never give the support that a bony one gives.

Fourthly, the method of an open operation and sewing up the fibrous covering and split lateral expansion of the quadriceps by buried sutures. This has, to my mind, little to recommend it in comparison with wiring, as the operation would be as severe, and the result not so secure.

Fifthly, wiring. It is usual to make a longitudinal incision and then carefully to scrape away all material between the fragments, to bore and pass the wire, then to irrigate the joint, washing out all clot, then tighten the wire. I always put a drainage tube into the joint on the outer side, and remove it in twenty-four hours. One can generally rely upon a single stout silver wire. A good plan, when the lower fragment is small, is to pass two wires through one hole in the lower, and separate them to pass through two holes in the upper. This gives a very firm hold. The method was suggested by my colleague, Mr. Paul Bush.

Wiring the patella should not be done too soon after the accident, the joint should be allowed at least forty-eight hours to begin to recover from the immediate effect of the injury. I have seen a disastrous result from the operation being done on the same day as the fracture. Certainly the operation should not be undertaken by any one who has not complete confidence in his antiseptics and in his carpentering skill, nor in cases where the general health is not good; but as far as my experience and my ideas go, it is the right thing to recommend in young adults who have to use their legs in earning their living, or who wish to be able to indulge in a fair amount of active exercise.

I briefly mention two special cases:—

Case 1.—F. A., aged 40. When playing hockey on November 27th, 1897, slipped and sustained a transverse fracture of the left patella.

November 30th. Patella wired with a single silver wire, joint washed out with boracic lotion and drained. Wound healed well.

Sixteen days later passive movements were begun. Patient went home January 7th, six weeks after the operation.

He played cricket all the following summer, and on September 10th, 1898, whilst bowling in a match the right knee-cap gave way. He was found to have a transverse fracture just below the middle.

September 19th. Right patella wired—one silver wire (*Vide* skiagram, Fig. 2.)

October 11th. Twenty-two days after operation passive movement was commenced—about fifteen degrees from horizontal.

Patient went out well six weeks from the operation, well able to bend both knees.

He is playing cricket this season.

Case 2.—C. W. In December, 1892, fractured left patella. This was treated at the Bristol Royal Infirmary with back splints and cross-bars, the fragments being pulled together by strapping. He wore a leather splint for some months. Six months later he was readmitted for stretching of the union, and was treated as on the previous occasion.

March 2nd, 1899, was admitted for a similar accident to the same patella—the interval between the fragments was fully one inch. (*Vide* skiagram, Fig. 3.)

March 14th. I cut away the dense fibrous tissue between the fragments, and freshened the ends of the bones and brought them together with two wire sutures passed through one hole in the lower and two holes in the upper fragment. (*Vide* skiagram, Fig. 4.)

March 29th. Wounds were quite healed—passive movements were begun sixteen days after operation.

April 24th. Patient went out, able to walk easily without a splint, bending his knee fairly well with no separation of fragments.

Fig. 1.

Fig. 2.

Fig. 3.

Fig. 4.

## Figures and Tables

**Fig. 1. f1:**
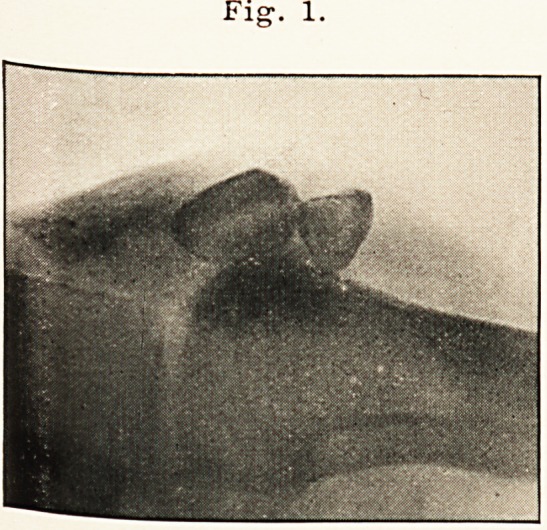


**Fig. 2. f2:**
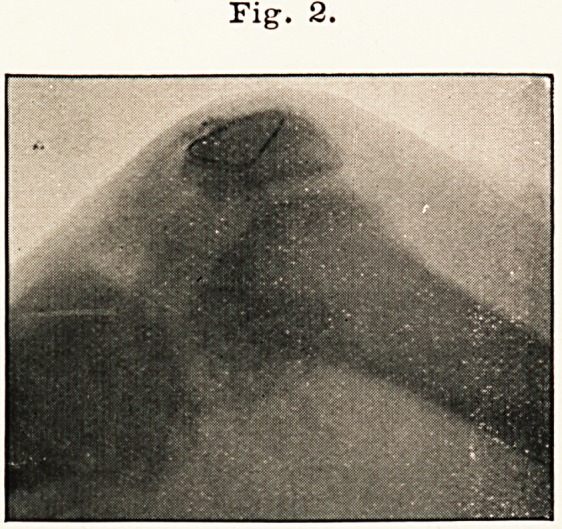


**Fig. 3. f3:**
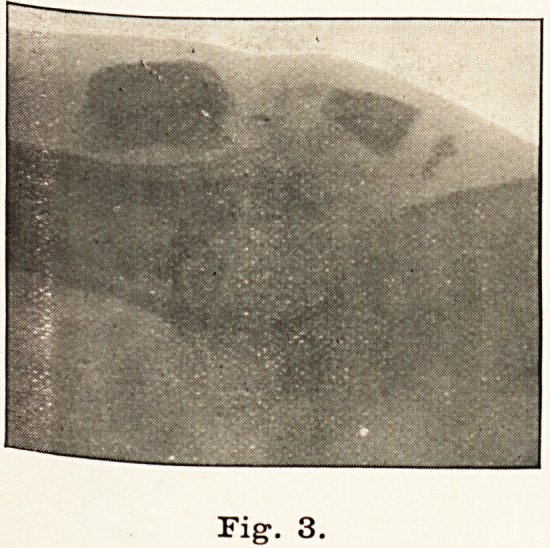


**Fig. 4. f4:**